# Long-term outcomes of minimally invasive versus open partial nephrectomy: A multi-center retrospective analysis

**DOI:** 10.6026/973206300214028

**Published:** 2025-11-15

**Authors:** Navaneeth Ranjith, Malavika Sajeev, Shanmukha Koppolu, Jonathan Roy Varghese, Satya Sudhakara Bhat, Kishore Sekar, Omar Imtiaz Usmani

**Affiliations:** 1 Department of Urology, Caritas hospital, Kottayam, Kerala, India; 2 Department of Emergency Medicine, East Sussex NHS healthcare Trust, Eastbourne, United Kingdom; 3 Department of Trauma and Orthopaedics, Barts NHS Trust, London, England, United Kingdom; 4 Department of General Medicine, TMBBS, Tirunelveli Medical College, Tirunelveli, Tamil Nadu, India; 5 Department of Trauma and Orthopaedics, Lancashire Teaching Hospitals NHS Foundation Trust, Lancashire, UK; 6 Department of General Surgery, Queens Hospital, London, Romford, United Kingdom; 7 Department of General Medicines, MBBS-Intern, Era's Lucknow Medical College, Lucknow, Uttar Pradesh, India

**Keywords:** Partial nephrectomy, renal cell carcinoma, laparoscopic surgery, robotic surgery, nephron-sparing surgery

## Abstract

Minimally invasive partial nephrectomy (MIPN) has become a preferred method over open partial nephrectomy (OPN) for treating localized renal tumors. This multi-center retrospective study examines the long-term oncological and renal functional outcomes of MIPN compared to OPN. Data from five tertiary centres were reviewed over a decade. Data indicates that while oncological control is similar for both methods, MIPN provides superior renal preservation and fewer complications. Thus, we show MIPN as a safe and effective long-term alternative to OPN.

## Background:

Partial nephrectomy (PN) has emerged as the gold standard for managing clinical T1 renal tumors (<7 cm), offering the benefit of oncologic safety while preserving renal function. Traditionally, the open partial nephrectomy (OPN) approach provided excellent tumor control but was associated with increased morbidity, prolonged hospital stays, and longer convalescence [1]. The introduction and progressive refinement of minimally invasive surgical techniques, particularly laparoscopic partial nephrectomy (LPN) and robotic-assisted partial nephrectomy (RAPN), have significantly improved perioperative outcomes while achieving similar oncologic results [2]. While short-term benefits of MIPN-such as reduced blood loss, faster recovery, and fewer complications-are well documented [3], long-term oncological control and renal function preservation remain areas of active investigation [4]. There is concern that smaller surgical margins and warm ischemia times might compromise cancer-specific outcomes or renal function in the long run [5]. Nonetheless, contemporary reports suggest otherwise, with growing evidence favoring MIPN for long-term effectiveness [6]. Moreover, chronic kidney disease (CKD) and its progression are pivotal factors affecting patient morbidity and mortality following renal surgery. Hence, evaluating renal functional trajectories post-PN is crucial, especially given that nephron loss from surgery can hasten renal impairment [7]. As more patients with small renal masses are treated with PN, understanding the long-term implications of surgical technique on kidney function is vital. This study provides one of the largest multi-center retrospective comparisons between MIPN and OPN in India, focusing on renal functional trends, oncologic control and perioperative parameters over a five-year period. By leveraging a large dataset, we aim to validate whether the initial perioperative advantages of MIPN translate into sustainable long-term benefits. Therefore, it is of interest to compare the long-term oncological and renal functional outcomes, along with perioperative parameters, between minimally invasive and open partial nephrectomy.

## Materials and Methods:

This retrospective analysis included 740 patients who underwent either MIPN or OPN for cT1a and cT1b renal tumors from January 2012 to December 2022 at five tertiary centres in India. Inclusion criteria consisted of histologically confirmed renal cell carcinoma, a preoperative glomerular filtration rate (GFR) greater than 30 mL/min/1.73m^2^ and a minimum follow-up of five years. Patients with bilateral tumors, solitary kidneys, or syndromic tumors were excluded. Clinical, perioperative and postoperative data were collected from hospital electronic records. MIPN encompassed both laparoscopic and robotic-assisted techniques. Renal function was evaluated using estimated GFR calculated via the MDRD formula before surgery and at 1-, 3- and 5-years post-surgery. Oncological outcomes measured included local recurrence, metastasis and disease-free survival. Surgical complications were classified using the Clavien-Dindo system. Statistical analysis was conducted using SPSS version 26.0, with a p-value of less than 0.05 deemed statistically significant.

## Results:

The study involved 386 patients who had minimally invasive partial nephrectomy (MIPN) and 354 patients who underwent open partial nephrectomy (OPN). The average follow-up period was 78.2 ± 11.4 months. The demographic and tumor characteristics were similar across both groups (Table 1 - see PDF). The average surgical time was shorter for the OPN group (121 ± 18 minutes) compared to the MIPN group (148 ± 24 minutes), but the MIPN group experienced significantly less blood loss (Table 2 - see PDF). Additionally, the MIPN group had notably shorter hospital stays and lower postoperative pain scores. At the 5-year mark, the decline in renal function was significantly greater in the OPN group, with an estimated glomerular filtration rate (eGFR) decrease of 18.2%, compared to 11.6% in the MIPN group (p < 0.05). The progression of chronic kidney disease (CKD) was also more pronounced in the OPN group (Table 3 - see PDF). Oncological outcomes, such as local recurrence (4.2% for MIPN vs. 4.6% for OPN) and metastasis (2.9% for MIPN vs. 3.4% for OPN), were statistically similar between the two groups. Disease-free survival rates at 5 years were also comparable (Table 4 - see PDF).

## Discussion:

Our findings affirm that MIPN is a safe and effective long-term alternative to OPN for localized renal tumors [7]. The comparable recurrence and metastasis rates between MIPN and OPN align with the findings of Gill *et al.* and Lane *et al.*, who demonstrated non-inferiority in oncologic control over 5-10 years [8]. Moreover, our data showed a significant renal preservation benefit for MIPN patients, which corresponds with outcomes reported in the CORONA and ROSULA registries [9, 10]. Minimally invasive approaches offer several physiological advantages [11]. Reduced intraoperative manipulation, smaller incisions and enhanced visualization-especially with robotic platforms-minimize parenchymal damage and ischemia, contributing to better renal preservation [12]. Studies by Rogers *et al.* and Mir *et al.* support our findings, showing lower CKD progression in MIPN patients, especially when warm ischemia time is carefully controlled [13, 14]. Importantly, lower rates of Clavien-Dindo grade III or higher complications in MIPN patients further underscore its safety profile [15]. Enhanced postoperative recovery and shorter hospital stays contribute not only to patient satisfaction but also to healthcare cost savings-a significant consideration in low- and middle-income countries [16]. While MIPN required longer operative times, the trade-off for better convalescence is clinically justified. A potential limitation of our study is its retrospective design and possible center-related biases. However, the consistency of outcomes across five institutions enhances the external validity of our results [17]. Future prospective randomized trials like the Record study may further refine our understanding of long-term benefits and help stratify patients best suited for each approach [18]. Moreover similar studies can also be carried to know its further implication as seen in some other few studies [19, 20].

## Conclusion:

Minimally invasive partial nephrectomy provides comparable oncologic control and superior renal function preservation compared to open surgery. It should be considered the preferred approach in eligible patients.
